# Cadmium Toxicity and Treatment

**DOI:** 10.1155/2013/394652

**Published:** 2013-06-03

**Authors:** Robin A. Bernhoft

**Affiliations:** Bernhoft Centers for Advanced Medicine, 11677 San Vicente Blvd, Suite 208/211, Los Angeles, CA 93023, USA

## Abstract

Cadmium is a heavy metal of considerable toxicity with destructive impact on most organ systems. It is widely distributed in humans, the chief sources of contamination being cigarette smoke, welding, and contaminated food and beverages. Toxic impacts are discussed and appear to be proportional to body burden of cadmium. Detoxification of cadmium with EDTA and other chelators is possible and has been shown to be therapeutically beneficial in humans and animals when done using established protocols.

## 1. Introduction

Cadmium (Cd) is a naturally occurring metal situated in the Periodic Table of the Elements between zinc (Zn) and mercury (Hg), with chemical behavior similar to Zn. It generally exists as a divalent cation, complexed with other elements (e.g., CdCl_2_). Cd exists in the earth's crust at about 0.1 part per million [1], usually being found as an impurity in Zn or lead (Pb) deposits, and therefore being produced primarily as a byproduct of Zn or Pb smelting. 

Commercially, Cd is used in television screens, lasers, batteries, paint pigments, cosmetics, and in galvanizing steel, as a barrier in nuclear fission, and was used with zinc to weld seals in lead water pipes prior to the 1960s. Approximately 600 metric tons are produced annually in the United States, and about 150 metric tons are imported [2]. 

Human exposure to Cd occurs chiefly through inhalation or ingestion. Ten to fifty percent of inhaled cadmium dust is absorbed, depending on particle size. Absorption through skin contact is negligible. About five to ten percent of ingested Cd is absorbed, also depending on particle size. Intestinal absorption is greater in persons with iron, calcium, or zinc deficiency [3]. 

Cigarette smoking is considered to be the most significant source of human cadmium exposure [4]. Blood and kidney Cd levels are consistently higher in smokers than nonsmokers. Inhalation due to industrial exposure can be significant in occupational settings. for example, welding or soldering, and can produce severe chemical pneumonitis [3]. 

Cadmium exposure occurs from ingestion of contaminated food (e.g., crustaceans, organ meats, leafy vegetables, rice from certain areas of Japan and China) or water (either from old Zn/Cd sealed water pipes or industrial pollution) and can produce long-term health effects. Contamination of drugs and dietary supplements may also be a source of contamination [5]. 

## 2. Absorption and Distribution

After absorption, Cd is transported throughout the body, usually bound to a sulfhydryl group-containing protein like metallothionein. About 30% deposits in the liver and 30% in the kidneys, with the rest distributed throughout the body, with a clearance half-life of twenty-five years [6]. The half life of cadmium in the blood has been estimated at 75 to 128 days, but this half life primarily represents deposition in organs, not clearance from the body [7]. Consequently, blood, hair, and urine Cd levels are poor surrogates for body burden and chiefly reflect recent exposure, as is also true with the other heavy metals. Accurate estimate of body burden of Cd will require urine provocation testing [8].

## 3. Mechanisms of Toxicity

Cadmium toxicity has been demonstrated in several organs, as discussed later. Cadmium induces tissue injury through creating oxidative stress [9–11], epigenetic changes in DNA expression [12–14], inhibition or upregulation of transport pathways [15–17] particularly in the proximal S1 segment of the kidney tubule [18]. Other pathologic mechanisms include competitive interference with the physiologic action of Zn or Mg [19–21], inhibition of heme synthesis [22], and impairment of mitochondrial function potentially inducing apoptosis [23]. Depletion of glutathione has been observed, as has structural distortion of proteins due to Cd binding to sulfhydryl groups [24]. These effects are magnified by interaction with other toxic metals such as Pb and As [25] and possibly ameliorated by Zn or Se (see later) and by factors increasing levels of Nrf2 [26, 27].

## 4. Clinical Toxicity

Clinical stigmata of cadmium toxicity depend on route, quantity, and rate of exposure. The chief organ of toxic impact in the human is the kidney, where the S1 segment of the proximal tubule is a major target of Cd deposition, with clinically observable defects in protein, amino acid, glucose, bicarbonate, and phosphate reabsorption (Fanconi syndrome) resulting from Cd-induced oxidative damage to transport proteins and mitochondria which may induce apoptosis of tubular cells [28–31]. Effective antioxidant therapies are being sought [32], and there is *in vitro *evidence that selenium [33] and zinc [34] may at least partially antagonize the toxic effects of cadmium. About 30% of body cadmium is deposited in the kidney tubule region, as discussed earlier, with tubular damage being proportionate to the quantity of cadmium not bound to metallothionein [35]. Diabetics are more susceptible to renal tubular damage from Cd exposure than controls [36]. 

Cadmium may also impair Vitamin D metabolism in the kidney [37], with deleterious impact on bone. This effect, coupled with direct Cd impairment of gut absorption of calcium and derangement of collagen metabolism, can produce osteomalacia and/or osteoporosis [3]. The most extreme example of this process is *itai-itai* disease in Japan, which combines severe pain from osteomalacia with osteoporosis, renal tubular dysfunction, anemia, and calcium malabsorption [38].

Mechanisms of Cd toxicity in bone include stimulation of fibroblast growth factor 23 which induces phosphaturia and decreases phosphate uptake, leading to osteomalacia [39]. Cd is toxic to MC3T3 osteoblasts by unknown mechanisms [40] and stimulates osteoclasts, thereby inducing osteoporosis [41]. Cd decreases serum osteocalcin levels in rats [42]. These factors apparently combine to induce calciuria, increase bone resorption and decrease bone mineral density in Cd-exposed children [43]. 

Cadmium affects the cardiovascular system in several ways. The literature is somewhat contradictory, but much of it supports a role for Cd in inducing hypertension [44] and diabetes [45], with apparent direct toxic impact on gene transcription in the vascular endothelium [46]. Epidemiological evidence links Cd with sudden cardiac death [47], peripheral arterial disease [48], increased vascular intima media thickness [49], and myocardial infarction [50]. Proposed mechanisms include disruption of calcium channels and direct vasoconstriction as well as inhibition of NO and possibly other vasodilators [51]. Cd also directly induces oxidative stress, increases lipid peroxidation and depletes glutathione [52–54]. Cadmium accumulates in the wall of the aorta [55]. Cadmium is apparently brought into the vascular wall by Cd-laden monocytes which differentiate into foam cells [56]. Cadmium is also deposited in vascular smooth muscle cells and produces apoptosis of endothelial cells [57]. Direct myocardial structural damage has also been documented [58]. 

Hematopoeisis is adversely affected, most notably in *itai-itai *disease where severe anemia is observed, in association with marked suppression of erythropoietin production [59]. Hemolysis may also be a factor in producing Cd-associated anemia, which may produce iron-deficient indices despite increased body Fe stores resulting from hemolysis and increased duodenal Fe absorption [60]. 

Similarly, the immune system suffers form Cd-induced impairment at several levels. Prenatal Cd exposure may impair postnatal T cell production and response to immunization [61], as well as dysregulated thymocyte development [62]. Post-natal Cd exposures induce cell cycle arrest and apoptosis in splenocytes [63]. Cd induces increased rates of autoimmunity, increased production of nonspecific antibodies, and decreased production of antigen-specific antibodies [64]. Lymphocyte proliferation and natural killer cell activity are also suppressed by Cd [65]. Metallothionein protects against Cd immune toxicity [66].

Cadmium has considerable endocrine disruption capacity, apparently disregulating all pituitary hormones [67]. In the 2007-8 NHANES survey, elevated blood Cd levels were associated with suppressed TSH production, while increased urine Cd was associated with elevated serum levels of T3 and T4 [68]. 

Cadmium is considered to be a metalloestrogen, but evidence to support that contention is stronger in *in vitro* and *in vivo *animal studies than in population-based human studies [69]. It is based partly on binding of Cd to breast cancer estrogen receptors [70]. It seems that estrogen-like effects of Cd result from a mechanism different from that of steroidal estrogens [71]. 

Male infertility in rats from Cd exposure is due to damage to the blood-testis barrier, decreasing germ cell adhesion leading to germ cell loss, reduced sperm count and subfertility or infertility [72]. Rat studies further suggest Cd may induce production of prostaglandin F2alpha which causes cavernosal vasoconstriction and suppressed testosterone synthesis and secretion in the male, as well as destruction of corpus luteum and fetus in the female. These occur perhaps through inhibition of steroidogenic acute regulatory protein (StAR) which is responsible for the rate limiting step in steroidogenesis [73]. Human epidemiological studies have not, however, supported Cd as a cause of male infertility or erectile dysfunction. 

Cadmium exposure is a known risk factor for developing insulin resistance [74, 75]. In the Korean NHANES experience, there is a strong correlation between blood Cd and development of metabolic syndrome [76], the mechanisms of which remain unelucidated but may involve mechanical distortion of the insulin receptor. The Cd effect on insulin resistance may be minimized by supplementation of Fe, Ca, Mg, and Zn (which also decreases the Cd-associated risks of cancers, fractures, vascular disorders, and total mortality) [77].

Cadmium has been observed to cause oxidative stress and histologically visible membrane disturbances in the central nervous system, with reduction in acetylcholinesterase activity, increase in oxidative stress markers, depletion of glutathione, superoxide dismutase 2, and other antioxidants, and depletion of catalase, glutathione peroxidase, and glutathione-S-transferase [78]. These changes have apparently led to apoptosis of cortical cells in the central nervous system,possibly due to phosphorylation of calcium/calmodulin-dependent protein kinase II [79]. Cd can also inhibit influx through calcium channels [80].

Clinically, humans with elevated blood or urine Cd demonstrate decreased attention level and memory [81]. Additionally, humans with high urinary Cd levels had significantly decreased low-frequency hearing [82]. Similarly, rats with high urinary Cd exhibit decreased learning ability. Intranasal cadmium destroys olfactory nerve function in the rat [83]. Cadmium raises the frequency of spontaneous cortical electrical activity in the rat, lengthens the latency of sensory-evoked potentials, and impairs frequency following ability even in rats without detectable Cd brain deposition [84].

The United States Environmental Protection Agency considers Cd to be a Class B1 carcinogen [85]. There is contradictory evidence linking Cd exposure to breast cancer [86–88] and denying that link [89]. Prostate cancer is also correlated with Cd consumption [90, 91] as is pancreatic cancer [92–94]. In the Third NHANES cohort, Cd was associated with pancreatic and lung cancer and non-Hodgkin's lymphoma [95]. Other investigators have found a plausible association between Cd and lung cancer [96–98] and weak evidence for a link between Cd and non-Hodgkin's lymphoma [99, 100]. 

## 5. Reduction of Body Burden

There is no agreement in the literature regarding treatment of Cd toxicity. Human studies are few and anecdotal. While clinical protocols exist for the use of EDTA, DMPS, and DMSA [101–104], they rely for the most part on clinical experience and on *in vitro* and animal studies [105, 106]. EDTA is the agent most widely accepted for clinical use. While it may seem axiomatic that reduction of body Cd burden would decrease its toxic effects, not all authorities agree that active measures beyond avoidance are indicated, at least for acute poisoning, where concern exists that chelation may aggravate damage to the kidney tubules [107, 108]. For chronic exposures, however, there is considerable evidence of chelation's clinical efficacy, in humans and in experimental animals. Several chelators have been used. Clinically available chelators include EDTA, DMPS, DMSA, and British Anti-Lewisite (BAL). BAL is more toxic than its derivatives, DMPS and DMSA, and is seldom used clinically. Several experimental chelators, including DTPA [109] (available from the National Strategic Reserve for radiation poisoning), NaB [110], and others [111, 112], are also being investigated but are not clinically available at present. 

It is clear that EDTA [113, 114], DMPS [115], and DMSA [116] increase urinary excretion of Cd, but DMSA seems to have little impact on overall body burden of Cd [117, 118]. Studies *in vitro* [119] and *in vivo* [120] suggest that EDTA is superior to DMSA in mobilizing intracellular Cd. In clinical use, EDTA is credited with an anecdotal report of relief of rheumatoid arthritis [121], as well as reduction of oxidative stress [122], and reduction of general metal toxicity [123, 124]. The efficacy of EDTA is apparently improved with concomitant use of glutathione [125] which also protects against nephrotoxicity; efficacy may also be improved with concomitant use of antioxidants [126] including mannitol [127], as well as thiamine [128], methionine [129], or zinc [130]. DMPS has not been studied as extensively as EDTA and DMSA but appears effective in rats [131], is available over the counter in Germany, and may be compounded legally in the United States.

EDTA is approved by the FDA for lead and other heavy metals, and has a long history of safe use. It should not be given faster than one gram per hour nor in dosage greater than three grams per session. Sessions should be at least five days apart, and replacement of essential minerals should be done orally between sessions. Several effective protocols exist implementing these principles [101–104]. 

Cd is also significantly present in sweat during sauna, which appears to be a moderately successful modality for reducing body burden of Cd without risk of tubular damage [132], albeit at a rate slower than that of intravenous chelation with EDTA.

## 6. Conclusion

According to the Third National Report on Human Exposure to Environmental Chemicals (NHANES), Cd exposure is widespread in the general population [133]. No standards exist correlating blood or urine Cd measurements with clinical toxicity; so, no conclusions are drawn on the significance of blood or urine levels. This is also true since blood and urine levels do not correlate with body burden, as discussed earlier. Given the ubiquity of Cd in the environment, the multisystem toxicity of Cd as discussed previous, and the generally benign nature of EDTA treatment administered under any of the aforementioed clinical protocols, it would seem reasonable to screen high risk individuals (smokers, persons with industrial exposures, etc., as above) and those with potential clinical indications and treat those with elevated Cd levels on provocation. 
